# Sputtering-Assisted Synthesis of Copper Oxide–Titanium Oxide Nanorods and Their Photoactive Performances

**DOI:** 10.3390/nano12152634

**Published:** 2022-07-30

**Authors:** Yuan-Chang Liang, Tsun-Hsuan Li

**Affiliations:** Department of Optoelectronics and Materials Technology, National Taiwan Ocean University, Keelung 20224, Taiwan; chris6080624@gmail.com

**Keywords:** microstructure, composites, photoactivity

## Abstract

A TiO_2_ nanorod template was successfully decorated with a copper oxide layer with various crystallographic phases using sputtering and postannealing procedures. The crystallographic phase of the layer attached to the TiO_2_ was adjusted from a single Cu_2_O phase or dual Cu_2_O–CuO phase to a single CuO phase by changing the postannealing temperature from 200 °C to 400 °C. The decoration of the TiO_2_ (TC) with a copper oxide layer improved the light absorption and photoinduced charge separation abilities. These factors resulted in the composite nanorods demonstrating enhanced photoactivity compared to that of the pristine TiO_2_. The ternary phase composition of TC350 allowed it to achieve superior photoactive performance compared to the other composite nanorods. The possible Z-scheme carrier movement mechanism and the larger granular size of the attached layer of TC350 under irradiation accounted for the superior photocatalytic activity in the degradation of RhB dyes.

## 1. Introduction

TiO_2_ nanorods are widely used as template for fabrication of photoexcited devices [1]. However, the main drawback of the intrinsic properties of TiO_2_ is its large energy gap, which means that it only absorbs light in the ultraviolet region. Recent progress on coupling the heterogeneous structure of TiO_2_ with visible light sensitizers has been demonstrated as a promising approach to substantially improve the light harvesting ability of the TiO_2_ template. Several binary visible-light sensitizers, such as Bi_2_O_3_, Cu_2_O, CuO, Fe_2_O_3_, CdS, and Bi_2_S_3_, have been adopted for coupling with TiO_2_ templates to achieve improved photoactive performance [2,3,4,5,6,7,8]. Among these visible-light sensitizers, binary oxides provide a better, more suitable process and chemical compatibility for integration with TiO_2_ templates in comparison with most sulfides. Notably, in comparison with n-n heterostructures, the construction of p-n heterostructures is a more promising approach for the enhancement of the photoactivity of TiO_2_-based composites. The p-n junction generates an internal electric field that can effectively suppress the recombination of photogenerated carriers in the composite system [5,9]. In addition, in terms of charge transport mode, the Z scheme often appears in organic degradation, CO_2_ reduction and photoelectric catalytic water splitting in heterostructured systems [10]. 

Among the various p-type visible-light sensitizers, copper oxides are distinguished by having diverse crystallographic phases and tunable band energy. Copper oxides are non-toxic and low cost materials rich in earth elements. Due to their low energy gap values, they have high optical absorption properties, resulting in excellent photoelectrochemical (PEC) performance and high energy conversion efficiency [11,12]. Recent work on the attachment of copper oxides onto TiO_2_ to enhance photoactive performance has attributed this improvement to the formation of a p-n junction. For example, electrodeposition of p-type Cu_2_O onto TiO_2_ nanoarrays improved the light absorption capacity and enhanced the photocatalytic activity [13]. Furthermore, p-type CuO nanoparticles attached onto TiO_2_ nanosheets effectively enhanced the photocatalytic activity for the oxidation of methanol to methyl formate [14]. CuO–Cu_2_O co-coupled TiO_2_ nanomaterials synthesized through chemical reduction and hydrolysis presented better charge separation rates and photocatalytic activity than those of pristine TiO_2_ [15]. The above examples show that attachment of single CuO or Cu_2_O or dual phase CuO–Cu_2_O onto parent TiO_2_ induces the formation of a p-n heterojunction between the copper oxide and the TiO_2_, resulting in the composites possessing an internal electric field and suppressing the recombination of photogenerated carriers. These phenomena can effectively increase the photocatalytic ability of the pristine TiO_2_. However, most investigations of the photoactivity of copper oxide–TiO_2_ composite systems are based on a fixed decorated oxide phase (one of the following: CuO, Cu_2_O, or CuO–Cu_2_O); this is attributed to the fact that precise manipulation of the crystallographic phase of copper oxide is still highly challenging using most chemical or physical synthesis routes. Systematic investigations of the effects of phase evolution on the photoactivity of copper oxide–TiO_2_ nanocomposite rods are still limited in number, and such information is an important reference for the design and tuning of the photoactive performance of copper oxide–TiO_2_ nanocomposites.

Thin copper oxide films can be synthesized via diverse chemical and physical routes [16,17,18]. Physical deposition of thin copper oxide films with adjustable crystallographic phases is a promising approach to design copper oxide–TiO_2_ nanocomposites with desirable photoactive performance for photoexcited device applications. It has been shown that the formation temperature of the crystalline copper oxide has profound effects on the crystallographic phases of the as-synthesized copper oxides [18,19]. However, such temperature-dependent copper oxide phase evolutions are not always similar between different studies because of the different copper oxide precursors initially formed and the different process parameters or routes used [20,21]. For example, a copper film was transformed into the Cu_2_O phase after annealing at 250 °C under an atmospheric environment for 1 h. Moreover, a mixed phase of Cu_2_O–CuO appeared when the annealing temperature was set between 250–350 °C. Finally, the CuO phase could be obtained with an annealing temperature above 350 °C [22]. In this study, a thin metallic copper film was sputter-coated onto a TiO_2_ nanorod template. The crystallographic phase of the copper oxide layer formed by postannealing the pre-deposited copper film was tuned to manipulate the photoactive performance of the copper oxide-decorated TiO_2_ nanorod composites. The approach used by this work to produce copper oxide-decorated TiO_2_ composite nanorods differs from previous reference works [13,14,15]. Most copper oxide-decorated TiO_2_ is synthesized through chemical routes. It is difficult to manipulate the copper oxide crystalline phase using these routes. Only one copper oxide phase is attached onto the TiO_2_ template. In contrast, by combining a sputtering process and postannealing procedures in this work, we could easily design different copper oxide crystal phases on the TiO_2_ templates. The correlation between the composition phase, microstructure, and photoactivity of the copper oxide layer attached onto the TiO_2_ nanorod template was systematically investigated. The results presented herein are important references for the design of copper oxide–TiO_2_ composite systems with desirable photoactivity for photoexcited device applications.

## 2. Experiments

The preparation of TiO_2_ composite nanorods decorated with a copper oxide layer can be divided into two steps. The first step was to prepare TiO_2_ nanorod arrays on F-doped SnO_2_ glass substrates. The detailed preparation procedures have been described elsewhere [23]. The second step included modification of the surfaces of TiO_2_ nanorods with a copper oxide layer by sputtering. A metallic copper disc with a size of 2 inches was used as the target. The metallic copper film was sputter-coated onto the surfaces of TiO_2_ nanorods at room temperature under a pure argon atmosphere. The working pressure was 20 mtorr, and the sputtering power was fixed at 30 W. The sputtering duration was 12 min. The as-synthesized metallic copper layers on the TiO_2_ nanorods were further subjected to an atmospheric annealing treatment for 1 h. The annealing temperature was varied between 200, 300, 350, and 400 °C to induce the formation of copper oxide from the metallic copper layer. The sample codes for the composite nanorods formed after 200, 300, 350, and 400 °C annealing were TC200, TC300, TC350, and TC400, respectively. 

The crystallographic structures of the various samples were characterized with grazing incidence angle X-ray diffraction (GID; BRUKER D8 SSS, Karlsruhe, Germany) using monochromatic Cu-K*α* radiation. A field emission scanning electron microscope (SEM; JSM-7900F, JEOL, Tokyo, Japan) equipped with an energy-dispersive X-ray spectrometer (EDS) was used for further investigations into the morphology and elemental distribution of the samples. A high-resolution transmission electron microscope equipped with EDS (HRTEM; Philips Tecnai F20 G2) was used to investigate the detailed structure and composition of the composite nanorods. An X-ray photoelectron spectroscopy (XPS ULVAC-PHI, PHI 5000 VersaProbe, Chigasaki, Japan) with Al Kα X-rays was used to detect the element binding states of the samples. The optical absorption spectral information for the samples was obtained with a UV-vis spectrophotometer (Jasco V750, Tokyo, Japan). Photoelectrochemical (PEC) performance and electrochemical impedance (EIS) were measured using a potentiostat (SP150, BioLogic, Seyssinet-Pariset, France). In the photoelectrochemical system, the effective area of the working electrode was 1.0 cm^2^. The reference and counter electrodes were Ag/AgCl (in saturated KCl) and platinum wire, respectively. A 0.5 M aqueous Na_2_SO_4_ solution was used as the electrolyte in the measurement system. During the photoexcitation experiments, a 100 W xenon lamp was used as the light source. Rhodamine B (RhB) solution (10^−5^ M) was used as the target pollutant for photodegradation experiments, and residual RhB concentrations after different degradation durations were estimated using a UV-vis spectrophotometer.

## 3. Results and Discussion

**Figure 1:**
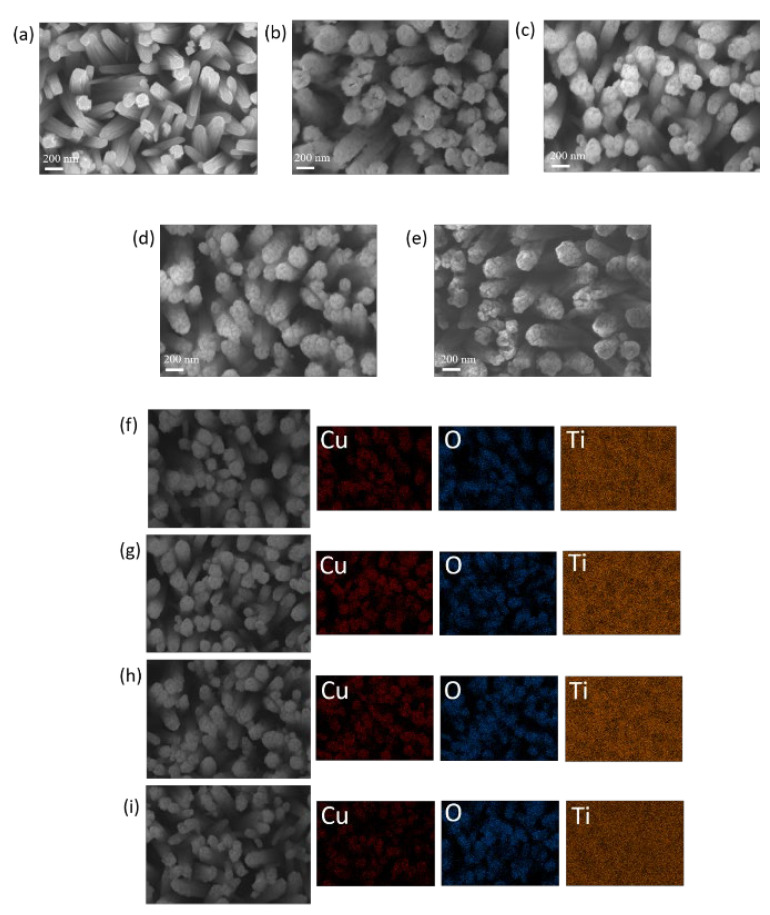
SEM images: (**a**) TiO_2_, (**b**) TC200, (**c**) TC300, (**d**) TC350, and (**e**) TC400. Corresponding Cu, O, and Ti mapping images of the composite nanorods: (**f**) TC200, (**g**) TC300, (**h**) TC350, and (**i**) TC400.

Figure 1a shows SEM micrographs of TiO_2_ nanorod templates. The TiO_2_ nanorods have rectangular cross-section morphologies and smooth sidewalls. Figure 1b shows an SEM micrograph of a TiO_2_ nanorod template coated with thin Cu films and postannealed at 200 °C. In comparison with the diameter of pristine TiO_2_ nanorods, it can be seen that the diameter of the TiO_2_ nanorods increased after copper coating and annealing at 200 °C. Furthermore, the morphology of the decorated layer wrapped in the outer layer of the TiO_2_ was film-like, and the sidewalls of the TC200 became rough. When the annealing temperature increased to 300 °C, the morphology of TC300 differed from that of TC200. The continuous film-like decorated layer of the composite nanorods formed with the lower annealing temperature of 200 °C transformed into a layer consisting of numerous tiny particles for the composite nanorods annealed at 300 °C (Figure 1c). As the annealing temperature was further increased to 350 °C, a clearer granular surface morphology was observed in the decorated layer for TC350 (Figure 1d). The surface morphology of the decorated layer transformed from having small particle features initially into larger granular features with the increase in temperature from 300 to 350 °C. Notably, when the annealing temperature reached 400 °C, the surface granular crystals of TC400 were further coarsened and aggregated, as revealed in Figure 1e. In a high temperature environment, the rapid formation of crystal nuclei leads to nucleus aggregation between the crystal nuclei and the coalescence of the crystal nuclei might occur. Furthermore, from a thermodynamic point of view, the aggregation of surface particles and the growth of crystallites decrease the surface energy to a stable condition. These factors account for the coarser surface granular features in TC400 [24,25]. The corresponding SEM-EDS mapping images of the TC composite nanorods are presented in Figure 1f–i. The Cu and O compositional distribution, which presented the appearance of a column shape, is visibly displayed in all SEM-EDS mapping images, preliminarily revealing the copper oxide layer homogeneously decorated onto the TiO_2_ nanorods after the copper film coating and postannealing procedures. In contrast, the Ti signal is distributed over a large area in the elemental mapping image and is not in a distinguishable column shape; this might be associated with the underlying effect of the TiO_2_ nanorod template on the TC composite nanorods. The EDS analysis demonstrated that the Cu/Ti atomic ratio of the representative sample (TC350) was 0.22.

**Figure 2:**
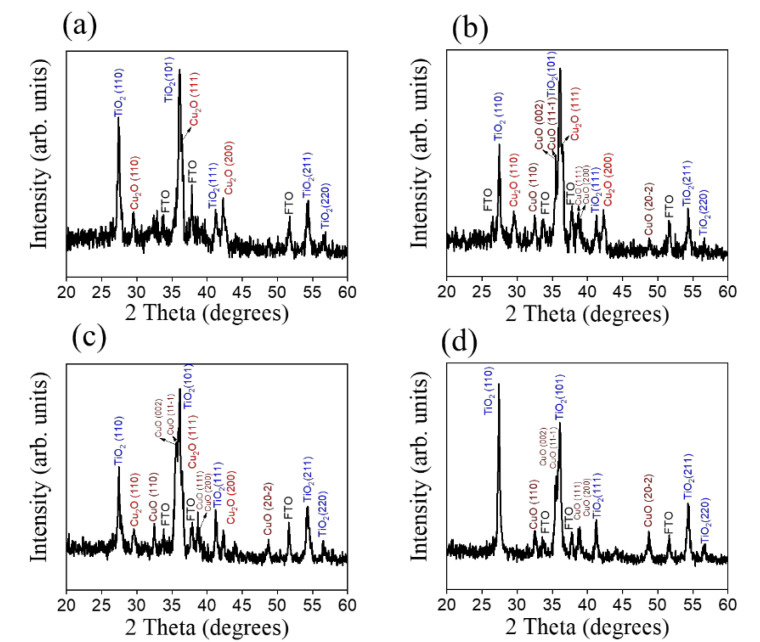
XRD patterns: (**a**) TC200, (**b**) TC300, (**c**) TC350, and (**d**) TC400.

Figure 2 shows the XRD patterns of various TC composite nanorods. In Figure 2, in addition to the Bragg reflection from the FTO substrate, several strong Bragg reflection peaks can be seen stably distributed at approximately 27.45°, 36.08°, 41.22°, 54.32°, and 56.64°, and they can be attributed to the (110), (101), (111), (211), and (220) crystal planes of the rutile TiO_2_ phase, respectively (JCPDS 0211276). Figure 2a shows the XRD pattern of the TC200. Three Bragg reflections centered at approximately 29.55°, 36.41°, and 42.29° can be observed. These Bragg reflection peaks can be attributed to the (110), (111), and (200) planes of cuprite Cu_2_O (JCPDS 05-0667), respectively. This confirms that the thin metallic copper film coated on the surfaces of the TiO_2_ nanorods was thermally oxidized to form cuprite Cu_2_O after annealing at 200 °C. This result is consistent with previous work on the full transformation of Cu thin films into cuprite Cu_2_O after a 200 °C atmospheric annealing procedure [21]. The high crystallinity of the Cu_2_O phase that appeared after the 200 °C atmospheric annealing procedure was a result of the easy binding of the copper atoms to oxygen atoms above 150 °C, which was mediated in accordance with Equation (1) [26]:

(1) $$2\mathrm{Cu}+0.5O_{2}\to {\mathrm{Cu}}_{2}O$$

Figure 2b,c show the XRD patterns of the TC300 and TC350. Compared with Figure 2a, six additional Bragg reflections can be observed in Figure 2b,c. These Bragg reflections are centered at approximately 32.50°, 35.41°, 35.54°, 38.70°, 38.90°, and 48.71°. These definite peaks match the characteristic peaks of tenorite CuO (JCPDS 48-1548) and correspond to (110), (002), (11-1), (111), (200), and (20-2), respectively. The characteristic peaks of Cu_2_O and CuO coexist in Figure 2b,c, which proves that Cu_2_O was partially converted into CuO when the sample was annealed above 300 °C. This result is very similar to that obtained by Sh. R. Adilov et al. In their work, a CuO oxide phase began to form when metallic copper films were annealed at 280 °C; furthermore, when the temperature was raised to 350 °C, a more obvious mixed phase of Cu_2_O and CuO was obtained in their thin-film samples [27]. Comparatively, as the temperature was increased from 300 °C to 350 °C, the characteristic peaks of CuO became more intense, revealing improved CuO crystalline content and crystalline quality. Notably, the CuO layer initially formed on the thin-film samples would decline the further oxidation rate was increased due to the thickening of the oxide layer and the increased distance that ions have to diffuse. In order to keep the oxidation rate stable and control the copper oxide phase, the annealing temperature was further increased to 400 °C in this study. In Figure 2d, it can be clearly seen that a single, pure CuO phase replaced the coexisting Cu_2_O and CuO phases in the films when the annealing temperature was raised to above 400 °C. This is associated with the fact that the initially formed Cu_2_O phase is converted into a CuO phase at higher temperatures according to Equation (2) [28]:

(2) $${\mathrm{Cu}}_{2}O+0.5O_{2}\to 2\mathrm{CuO}$$

The evolution of the copper oxide phase above 400 °C described herein has also been observed in previous work on the annealing temperature-dependent phase transformation of chemically deposited copper oxide films [29].

**Figure 3:**
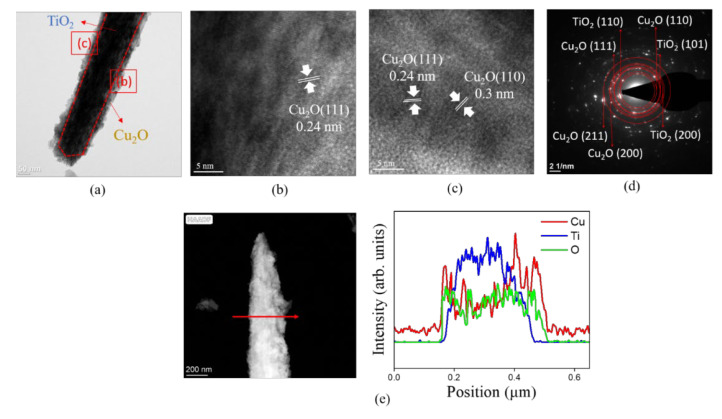
TEM analysis of TC200: (**a**) low-magnification TEM image; (**b**,**c**) HRTEM images of various regions of the composite rod as marked in (**a**); (**d**) SAED patterns of several TC200 nanorods; (**e**) EDS line scanning profiles across the composite rod.

Figure 3a shows a low-magnification TEM image of a single TC200 nanorod. The entire TiO_2_ nanorod was uniformly covered by a continuous Cu_2_O film. Rough and irregular surface features can be observed on the sidewalls of the nanorod. The decorated copper oxide layer thickness was estimated to be approximately 32 nm. The feature that appeared corresponded to the previous SEM observations. High-resolution (HR) TEM images of different regions of the TC200 nanorod are shown in Figure 3b,c. However, due to the repeated stacking of TiO_2_ and Cu_2_O, the lattice fringe arrangements in the inner region of the images cannot be easily distinguished. In contrast, clear lattice fringe arrangements can be observed in the outer regions of the HRTEM images, indicating the crystalline features of the decorated Cu_2_O layer. The spacing between these lattice fringes was measured to be approximately 0.24 nm and 0.3 nm in different orientations, and these lattice spacings corresponded to the interplanar spacings of the (111) and (110) planes of cuprite Cu_2_O, respectively [30]. Figure 3d shows selected area electron diffraction (SAED) patterns of several TC200 composite nanorods. It shows several diffraction spots arranged in concentric circles with different radii. These concentric circles correspond to rutile TiO_2_ ((110), (101), and (200)) and cuprite Cu_2_O ((111), (211), (110), and (200)). This confirms the formation of a crystalline Cu_2_O layer on the TiO_2_ nanorod. Figure 3e shows the cross-sectional EDS line-scan profiling spectra, in which the signal of Ti is distributed across the inner region of the nanorod, the signal of O is uniformly distributed over the entire nanorod region, and the signal of Cu is concentrated in the outer region of the nanorod. This indicates that the main core of the nanorod was TiO_2_ and the surface was covered with a layer of copper oxide. A TiO_2_ composite nanorod well-decorated with a Cu_2_O layer is visibly displayed.

**Figure 4:**
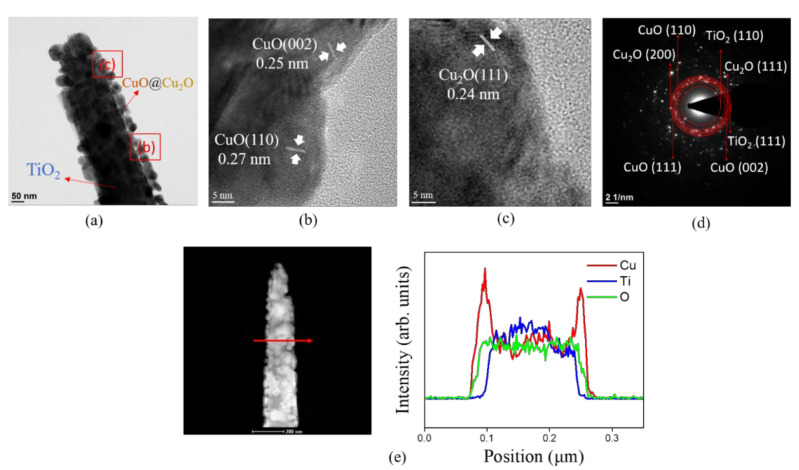
TEM analysis of TC350: (**a**) low-magnification TEM image; (**b**,**c**) HRTEM images of various regions of the composite rod as marked in (**a**); (**d**) SAED patterns of several TC350 nanorods; (**e**) EDS line scanning profiles across the composite rod.

**Figure 5:**
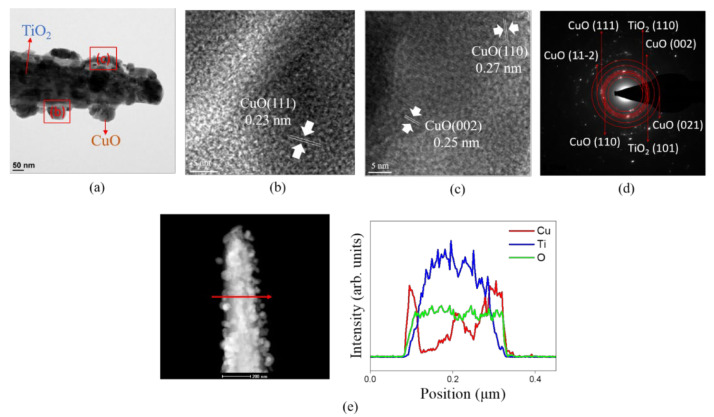
TEM analysis of TC400: (**a**) low-magnification TEM image; (**b**,**c**) HRTEM images of various regions of the composite rod as marked in (**a**); (**d**) SAED patterns of several TC400 nanorods; (**e**) EDS line scanning profiles across the composite rod.

Figure 4a shows a low magnification TEM image of a single TC350 nanorod. Compared with the TEM image of TC200 (Figure 3a), the copper film originally coated onto the surface of the TiO_2_ nanorod was transformed into a discontinuously decorated layer after the postannealing procedure. The discontinuous decorated layer consisted of numerous granular crystallites with a particle size of approximately 37 nm. Figure 4b,c present HRTEM images of different peripheral regions from Figure 4a. The decorated particles were further analyzed using HRTEM. The lattice fringes arranged with spacings of 0.25 nm, 0.27 nm, and 0.24 nm could be measured in the different orientations. They corresponded to the (002) and (110) crystal planes of the CuO phase and the (111) crystal plane of the Cu_2_O phase, respectively. The HRTEM images showed that the crystalline features of the Cu_2_O and CuO phases coexisted in the decorated discontinuous layer. This further confirmed the results for the previous XRD patterns. When the annealing temperature was raised above 300 °C, the original pure copper film was transformed into two different oxides, which coexisted on the surface of the TiO_2_ nanorods. Figure 4d shows the SAED patterns of multiple TC350 composite nanorods. The contributions of the crystallographic planes of cuprite Cu_2_O, tenorite CuO, and rutile TiO_2_ are visibly exhibited, proving that the ternary phases of Cu_2_O, CuO, and TiO_2_ coexisted in the TC350 nanorods. Figure 4e presents the cross-sectional EDS line-scan profiling spectra of the TC350 nanorods. The Cu signal was very strong in the outer region, and the Ti signal was mainly distributed in the inner region of the composite nanorod. The O signal was evenly distributed over the composite nanorod. A TiO_2_ composite nanorod well-shelled with copper oxide is demonstrated here, and the EDS analysis revealed that the Cu/Ti had an atomic ratio of 0.24. When the annealing temperature was further increased to 400 °C, as the low-magnification TEM image (Figure 5a) of the TC400 nanorod shows, the size of the particles wrapped over the sidewall surface of the TiO_2_ nanorod changed significantly compared to TC350 (Figure 4a). The size of the particles wrapped over the surface of the TiO_2_ nanorod was further increased to 55–70 nm. These seriously agglomerated particles on the TiO_2_ with relative large sizes can be attributed to the marked increase in the annealing temperature, which led to a substantially increased rate of nucleation and accelerated crystal size growth under the given annealing condition. During particle coalescence, the initially formed copper oxide particles could migrate to the TiO_2_ nanorod template surface and coalesce if motion yielded a reduction in overall system energy. Evidence for such a thermal annealing-induced Ostwald ripening process has been provided in other heterogeneous catalyst systems [31].

When the annealing temperature was further increased to 400 °C, as the low-magnification TEM image (Figure 5a) of TC400 nanorod shows, the size of the particles wrapped over the sidewall surface of the TiO_2_ nanorod changed significantly compared to TC350 (Figure 4a). The size of the particles wrapped over the surface of TiO_2_ nanorod was further increased to 55–70 nm. These seriously agglomerated particles with a relative large size on the TiO_2_ can be attributed to the marked increase in the annealing temperature, which led to a substantially increased rate of nucleation and accelerated crystal size growth under the given annealing condition. During particle coalescence, the initially formed copper oxide particles can migrate to the TiO_2_ nanorod template surface and coalesce if motion yields an overall system-energy reduction. Evidence for such a thermal annealing-induced Ostwald ripening process has been found in other heterogeneous catalyst systems [31]. Figure 5b,c show HRTEM images of the periphery of TC400 nanorod. Lattice spacings of 0.23 nm, 0.25 nm, and 0.27 nm in different orientations can be measured in Figure 5b,c, which corresponded to the interplanar distances of CuO (111), (002), and (110), respectively. These results confirm that the large-sized particles attached to the surface of TiO_2_ nanorod after annealing at 400 °C were CuO crystallites. Figure 5d shows the SAED patterns obtained from multiple TC400 composite nanorods. Obvious diffraction spots are arranged in concentric circles with different radii. Several crystallographic planes of CuO (111), (002), (11-2), (110), and (021) can be indexed in the SAED pattern. No other copper oxide phases were identified, indicating a TiO_2_-CuO composite structure for the TC400 nanorods. The TEM structural analyses showed the same results as revealed in XRD patterns. In addition, the cross-sectional elemental profiling spectra shown in Figure 5e also demonstrated a good compositional distribution for the copper oxide-decorated TiO_2_ nanorod composite structure. The TEM analysis results demonstrate that the annealing temperature effectively dominated the copper oxide phase and crystallite size on the TiO_2_ nanorod template.

**Figure 6:**
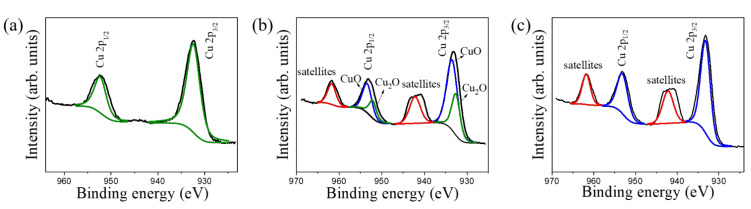
High-resolution XPS Cu 2p spectra: (**a**) TC200, (**b**) TC350, and (**c**) TC400.

Figure 6a displays the high-resolution Cu 2p XPS spectra for TC200. The distinct peaks centered at approximately 932.4 eV and 952.4 eV can be attributed to Cu 2p_3/2_ and Cu 2p_1/2_, respectively. The Cu binding energies matched the Cu^+1^ binding state in the Cu_2_O phase, and this was consistent with the results from a report on the XPS analysis of a sol-gel-derived thin Cu_2_O film [32]. Figure 6b shows the high-resolution Cu 2p XPS spectra for TC 350. The appearance of the XPS spectra is similar to that observed in a study on CuO@Cu_2_O heterostructures derived using the solvothermal method [33]. In contrast to the Cu 2p spectra for TC200, oscillating satellite peaks could be detected for TC350 at the binding energies of approximately 942.2 eV and 961.8 eV, which further indicated the existence of a CuO phase in TC350. This has also been demonstrated in the Cu 2p spectra analysis of pristine Cu_2_O and CuO thin films, in which pure Cu_2_O and CuO could easily be observed without and with the appearance of satellite peaks from the XPS spectra, respectively [32]. The spectra detected herein were further separated into several contributions. The intense fitted peaks located at 933.5 eV and 953.4 eV (blue line) were attributed to Cu 2p_3/2_ and Cu 2p_1/2_ of the CuO phase, respectively. There was a difference of approximately 20 eV between the Cu 2p_3/2_ and Cu 2p_1/2_ peaks of CuO, which matches well with the reported results for hydrothermally derived CuO nanoflowers [34]. In contrast, two relatively weak peaks (green line) appeared at 932.6 eV and 952.2 eV, corresponding to Cu 2p_3/2_ and Cu 2p_1/2_ of the Cu_2_O phase, respectively [35]. These results verify the coexistence of Cu_2_O and CuO phases in the decorated copper oxide layer in TC350. Figure 6c presents the high-resolution XPS spectra for Cu 2p in TC400. The distinct appearance of the satellite peaks (located at 942.2 and 961.8 eV) was observed (Figure 6c). The characteristic peaks centered at the binding energies of 933.3 eV and 953.3 eV corresponded to Cu 2p_3/2_ and Cu 2p_1/2_ of the CuO phase, respectively. These XPS results demonstrate that an adjustable copper oxide phase was obtained in the decorated copper film layer by varying the annealing temperature.

**Figure 7:**
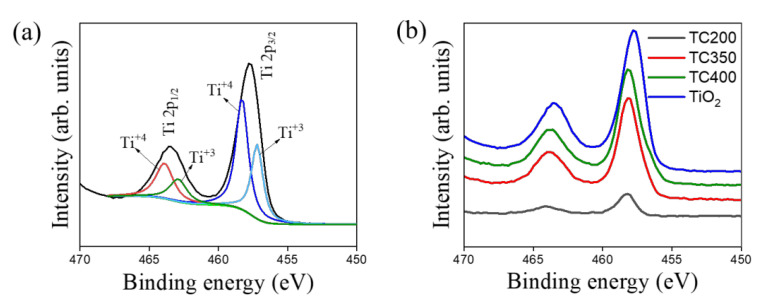
High-resolution XPS Ti 2p spectra: (**a**) TiO_2_ and (**b**) comparison of Ti 2p spectra of various samples.

Figure 7a shows the referenced Ti 2p core-level doublet spectra for the TiO_2_ nanorod template. The high-resolution XPS spectra were deconvoluted into four subpeaks. The more intense subpeaks at 458.3 eV and 463.9 eV corresponded to Ti 2p_3/2_ and Ti 2p_1/2_ for the Ti^4+^ valence state in TiO_2_, respectively. Furthermore, the subpeaks with weaker intensities and smaller binding energies of 457.2 eV and 462.9 eV corresponded to Ti 2p_3/2_ and Ti 2p_1/2_ in the Ti^+3^ valence state [36,37]. The presence of the mixed Ti^4+^/Ti^3+^ valance state indicates the possible presence of oxygen vacancies on the surfaces of the TiO_2_ nanorod template. Figure 7b shows a comparison of the Ti 2p core-level doublet spectra for TiO_2_, TC200, TC350, and TC400. It can be seen that the Ti 2p XPS spectra of the TC composite nanorods demonstrated positive shifts in binding energy positions in comparison with the binding energy position of pristine TiO_2_. The modification of TiO_2_ nanorods with copper oxides described herein might have changed the electronic state of Ti in Ti-O because of the formation of heterojunctions between the n-type TiO_2_ and p-type copper oxides. This has been demonstrated with CuO@TiO_2_ powders and core–shell N-TiO_2_@CuOx heterojunction composites formed using ball milling [38,39]. Notably, the Cu/Ti atomic ratio of TC350 was evaluated to be 3.6. The investigation depth of XPS is usually below 10 nm. This Cu/Ti atomic ratio substantially differs from the Cu/Ti atomic ratios calculated from the EDS spectra of electron microscopes because of the different measurement depths of the various analysis methods.

**Figure 8:**
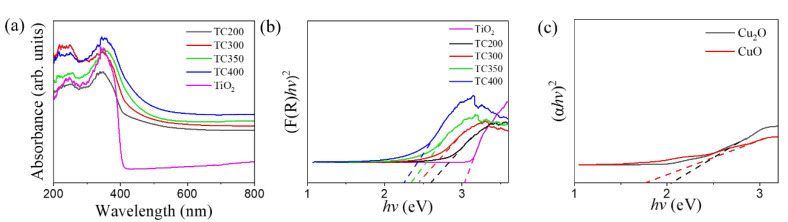
(**a**) Absorption spectra of various samples and Band-gap evaluations of (**b**) various nanorod samples, (**c**) pristine Cu_2_O and CuO films.

Figure 8a shows the optical absorption characteristics of pristine TiO_2_ nanorods and TC composite nanorods. A sharp absorption drop appeared at approximately 410 nm for TiO_2_ nanorod template, and this absorption edge was consistent with the inherent band-gap absorption of rutile TiO_2_ [1]. Notably, the TC composite nanorods demonstrated a significant red-shift extension of the absorption edge in comparison with that of the pristine TiO_2_. This can be attributed to the decoration of the TiO_2_ nanorods with Cu_2_O and CuO visible-light sensitizers. These visible-light sensitizers helped to absorb the longer wavelength spectra, making up for the inability of TiO_2_ to absorb visible light, and enhanced the absorption in the visible light region. The higher annealing temperature resulted in a larger size for the red-shift of the absorption edge of the TC composite samples; this was associated with the fact that the CuO formed at the higher annealing temperature had a narrower band-gap energy than that of Cu_2_O [40,41]. Figure 8b shows the Kubelka–Munk function (F(R)) vs. energy plots for various nanorod samples [42]. Notably, the TiO_2_ and copper oxides used herein were expected to exhibit a direct transition in the band-gap measurements. Therefore, the band-gap energy of the TiO_2_ nanorods and TC composite nanorods could be deduced from the (F(R)*hv*)^2^ vs. *hv* plots by extrapolating the straight portion of the curves to the energy axis. The TiO_2_ nanorod template was estimated to have an energy gap of approximately 3.03 eV. The energy gap values of TC 200, TC300, TC350, and TC400 were estimated to be approximately 2.59 eV, 2.43 eV, 2.34 eV, and 2.27 eV, respectively. The phase evolution of the decorated layer from Cu_2_O to CuO with increased annealing temperature visibly demonstrated a decreased energy gap in the TC composite nanorods. The band-gap energy variation in the copper oxides due to the phase evolution was consistent with a report on electrodeposited Cu_2_O/CuO powder oxides [43]. The UV-vis analysis demonstrated that the energy gap size of the TC composite nanorods could be effectively tuned by varying the postannealing temperature. In addition, the energy gaps of single CuO and Cu_2_O films were also estimated from the Tauc plot (Figure 8c). The energy gap values for CuO and Cu_2_O were estimated to be 1.76 eV and 2.04 eV, respectively, by extrapolating the curve tangent to the energy axis in Figure 8c. These values are similar to those from previous work on Cu_2_O formed with copper foil annealing and sputtering CuO [44,45].

**Figure 9:**
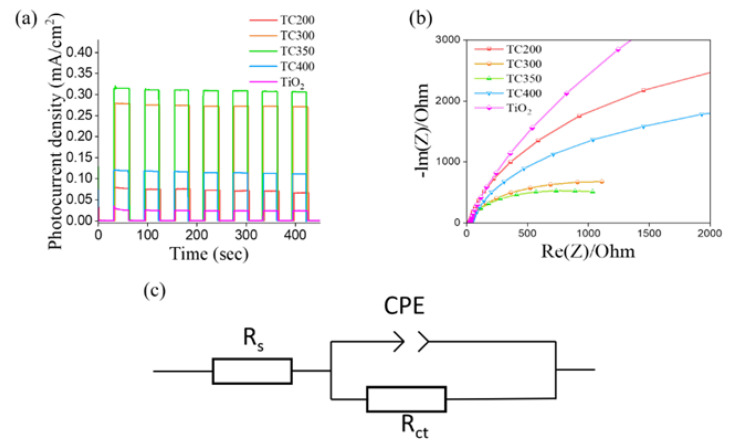
(**a**) Photocurrent density versus time curves for various samples at 1.2 V (vs. Ag/AgCl) under chopping illumination. (**b**) Nyquist plots for various samples under irradiation. (**c**) Possible equivalent circuits for Rct evaluation.

Figure 9a shows the transient photoresponses of various samples. Irradiation was applied with the full-band spectrum, and a bias potential of 1.2 V was used to measure the photocurrent. Photocurrent generation occurred entirely as a result of the on and off responses to the irradiation. Seven cycles of on/off irradiation were repeated, as shown in Figure 9a, and all samples could obtain a stable photogenerated current when the irradiation was turned on, indicating that the samples were stable under cycling chopping irradiation. A higher photocurrent indicates better efficiency for the separation of photogenerated charges and better photocatalytic activity for the photoelectrode [46]. Comparatively, all the TC composite nanorods exhibited improved photoresponses compared to that of the pristine TiO_2_. This was attributed to the fact that decoration with Cu_2_O and CuO visible-light sensitizers enhanced the light-harvesting ability of the TiO_2_ nanorod template, and the formation of heterojunctions in the composite system resulted in improved photogenerated carrier separation efficiency. Furthermore, compared to TC200, which had a single-phase Cu_2_O decoration, TC400 (with single-phase CuO decoration) had a higher photocurrent, which can be attributed to the narrower energy gap in CuO compared to Cu_2_O. This led to TC400 absorbing across a longer wavelength range than TC200, as revealed in the previous absorption analysis, thereby increasing light absorption and promoting the photoexcited carrier density. This has also been demonstrated in previous work on the photoactive performance of a Cu_2_O/CuO system [32,47]. Notably, TC300 and TC350 displayed the best photoresponse abilities among the various nanorod samples, revealing that the composite nanorod system decorated with dual Cu_2_O and CuO phases was a more efficient material combination for enhancing the photoactivity of the copper oxide–TiO_2_ composite nanorods. Figure 9b presents the Nyquist plots of various samples measured at the frequency range from 100 kHz to 0.1 Hz and a potential amplitude of 10 mV. The radius of the semicircles in Nyquist plots is associated with the interfacial charge transfer resistance [48]. Notably, TC350 had the smallest semicircular radius, and the pure TiO_2_ nanorod template exhibited the largest semicircular radius, indicating that TC350 had the smallest charge transfer resistance and TiO_2_ the largest. The sizes of the semicircle radii from the Nyquist plots for various samples were ordered in the following trend: TiO_2_ > TC200 > TC400 > TC300 > TC350. This result was also found with the transient photoresponse measurements. The multi-interface heterostructures consisting of TiO_2_, CuO, and Cu_2_O in TC350 and TC300 effectively helped to enhance the separation and transfer abilities of electron–hole pairs, as revealed in the previous I-t curves (Figure 9a). Similar coexistence of ternary phases leading to substantial improvements in PEC properties has also been demonstrated in BiVO_4_/CdS/CoOx core-shell composites [49]. These improvements can provide an opportunity to induce electron redistribution and synergistic effects at the interfaces for heterogeneous catalysis consisting of two or more components connected by well-defined interfaces [50,51]. The existence of multiple heterointerfaces in ternary phase composites improves their PEC properties. The charge transfer resistance can be estimated by fitting the arc radius of the Nyquist curves according to the proposed equivalent circuits in Figure 9c. Rs, CPE, and Rct represent the series resistance, constant phase element, and charge transfer resistance, respectively. Similar equivalent circuits have also been used in a ternary Fe_2_O_3_–MoS_2_–Cu_2_O nanofilm system to determine the Rct [52]. The representative fitting parameters for TC350 were Rct = 582 Ohm and Rs = 43.89 Ohm. After fitting the Nyquist plots using the proposed equivalent circuits, the Rct values for the other samples, TiO_2_, TC200, TC300, and TC400, were 3653, 1832, 702, and 1284 Ohm, respectively. Notably, although the TC300 and TC350 were both ternary-phase composite nanorods, lower interfacial charge transfer resistance in TC350 was observed in comparison to that of TC300. This might have been associated with the fact that, as the annealing temperature increases, the crystallite size of the decorated copper oxides increased, and this could have reduced the grain boundaries in the decoration layer. Therefore, TC350 had a better charge transport ability than TC300, and this was evidenced in the Rct.

**Figure 10:**
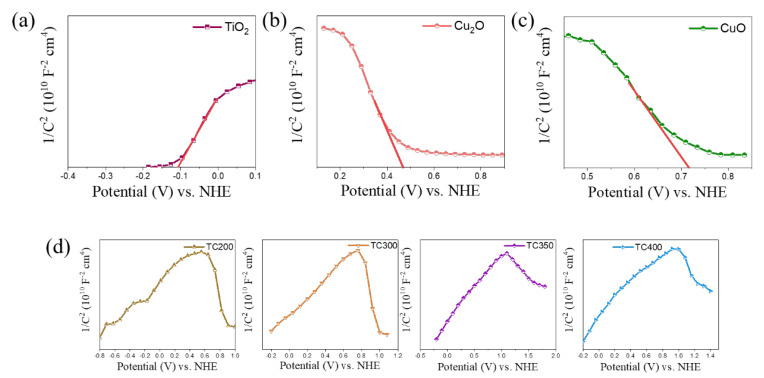
Mott–Schottky plots for various samples: (**a**) TiO_2_, (**b**) Cu_2_O, and (**c**) CuO. (**d**) A series of M−S plots for various composite nanorods.

In order to further analyze and construct the energy-band structure of the composite nanorods, measurements of the flat-band potential of the TiO_2_ nanorod template, Cu_2_O film, and CuO film were carried out and presented in Figure 10a–c. The M-S curves exhibited a positive slope for the TiO_2_ and negative slopes for the Cu_2_O and CuO, revealing the n-type nature of the TiO_2_ and the p-type nature of the Cu_2_O and CuO. According to the M-S equation [53], when 1/C^2^ is extrapolated to a value equal to 0, the X-axis intercept is equal to the flat-band potential of the material [54]. The flat-band potential of pure TiO_2_ was estimated to be about −0.11 eV (vs. NHE). The flat-band potential in n-type semiconductors is closer to the conduction band (CB) and the CB position of an n-type semiconductor is generally more negative (0.1 eV) than the flat-band potential [55]. After calculation, it was deduced that the CB of TiO_2_ was −0.21 eV. In contrast, the flat-band potential of the p-type semiconductor is closer to its valence band (VB) [56,57]. The flat-band potentials of Cu_2_O and CuO were estimated to be approximately 0.46 eV and 0.71 eV, respectively, as shown in Figure 10b,c. The VB positions of Cu_2_O and CuO were further calculated to be 0.56 and 0.81 eV (vs. NHE), respectively. The VB positions assessed herein are close to previously reported results for Cu_2_O and CuO [58,59]. Figure 10d shows the M-S curves for various TC composite nanorods. Inverted V-shaped M-S curves were observed for the all composite nanorods, demonstrating that the composites had both n-type and p-type electronic properties and confirming the formation of p-n junctions in the TC composite nanorods [60]. Construction of p-n junctions in composite systems has been posited to be a sensible strategy to enhance photocatalytic activity. The formation of a p-n junction with space charge regions at the heterointerface could induce the electric field-driven diffusion of electrons and holes and further inhibit the recombination of photogenerated charges [54,61].

**Figure 11:**
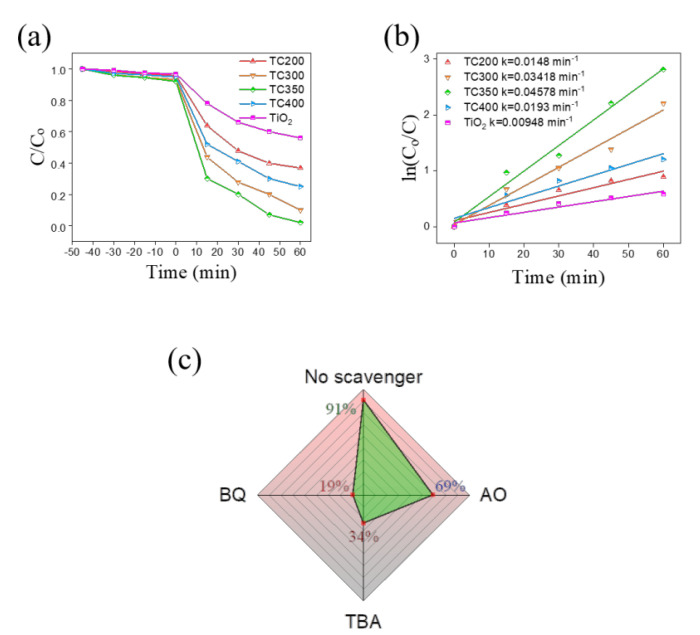
(**a**) C/Co vs. irradiation duration plots; (**b**) ln (Co/C) vs. irradiation duration plots; (**c**) degradation percentages of RhB solution with TC350 in the presence of various scavengers.

The photocatalytic ability of the samples was further estimated by using the formula whereby the percentage degradation = C/Co, where Co is the initial concentration of RhB solution and C is the time-dependent concentration of RhB solution upon irradiation. Figure 11a presents C/Co vs. irradiation duration plots for RhB solution with different samples. Adsorption–desorption equilibrium was reached by placing the photocatalysts in the RhB solution for 45 min in the dark before starting the photodegradation experiments. Under dark equilibration conditions for 45 min, the C/Co values for TiO_2_, TC200, TC300, TC350, and TC400 were approximately 3.1%, 4.2%, 6.8%, 7.9%, and 5%, respectively. This indicated that the TiO_2_ nanorod template decorated with copper oxide had an improved surface dye absorption capacity. After offsetting with a dark adsorption contribution, the degradation rates of TC200, TC300, TC350, and TC400 were approximately 59%, 83%, 90%, and 70%, respectively, with 60 min irradiation. The TC composite nanorods exhibited improved photodegradation abilities towards RhB solution in comparison to the pristine TiO_2_ nanorod template. Furthermore, among the various TC composites, TC350 had the highest photodegradation ability towards RhB solution under the given test conditions. In addition, the photodegradation kinetics of the RhB solution with all samples were also investigated and presented in Figure 11b. The pseudo-first-order kinetic equation is expressed as: kt = ln Co/C, where k represents the pseudo-first-order rate constant (min^−1^) for the initial degradation [61]. All the TC composite samples displayed larger k values than that of the pristine TiO_2_. Furthermore, TC350 had the highest k value of 0.04578 min^−1^. The photodegradation abilities of the photocatalysts towards organic pollutants were significantly related to the separation efficiency for electrons and holes. The magnitude trends for the k values for the various samples investigated herein were consistent with the previously measured PEC and EIS experimental results. In addition, the photocatalytic reaction was closely related to the active species produced in the process. The role of these species in the degradation reaction was investigated by measuring the variation in the degradation performance of the RhB solution with TC350 through the addition of various radical scavengers after 60 min irradiation. The radical capture experiments were performed using tert-butanol (TBA) as a hydroxyl radical (·OH) scavenger, ammonium oxalate (AO) as a hole quencher, and benzoquinone (BQ) as a superoxide radical (·O_2_^−^) scavenger. As shown in Figure 11c, when 1 mM AO was added, the RhB degradation efficiency slightly decreased to 69%, indicating that holes played a minor role in the degradation process. In contrast, adding TBA or BQ scavengers resulted in a more intense decrease in the photodegradation level of the RhB solution. This shows that ·O_2_^−^ and ·OH were the main radicals involved in the photodegradation process of the RhB solution with TC350. Comparatively, the removal of the ·O_2_^−^ active species resulted in the most significant decrease in the degradation efficiency.

**Figure 12:**
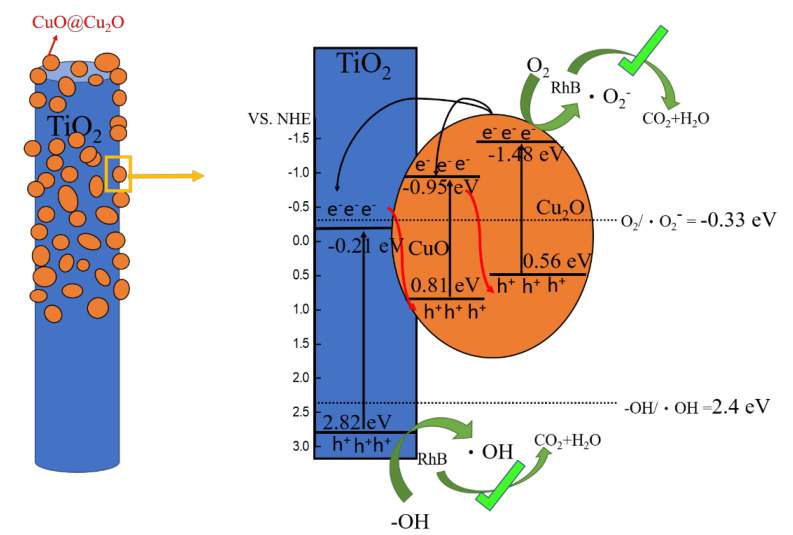
Possible mechanisms of charge transfer in TC350 under irradiation.

The band structures of pristine TiO_2_, Cu_2_O, and CuO were constructed according to the M-S measurements and the UV-vis analysis results, as shown in Figure 12. As shown in the previous scavenger experiments, the main active species involved in the TC350 photodegradation process with RhB solution were superoxide and hydroxyl radicals. Moreover, superoxide radicals demonstrated a greater contribution than hydroxyl radicals, as seen from the constructed band alignment in the ternary TC350 composite nanorods. If the electron–hole transfer route in the TC350 composite nanorods had followed the type II transfer mode, superoxide and hydroxyl radicals would not have been formed according to the relative band positions of the CB, VB, and redox potentials [62]. Therefore, none of the electrons/holes at the CB/VB positions would reach the required redox potential, so superoxide and hydroxyl active species would not have been produced with this mechanism. This contradicts the previous scavenger experiments. The Z-scheme mechanism shown in Figure 12 is more appropriate to explain the movement of photogenerated electrons/holes and the generation of active species for photodegradation. Under irradiation, photoinduced carriers form in the composite system (reaction 3). Through the movement of photogenerated carriers in the Z-scheme mechanism, the holes finally accumulated in the VB of TiO_2_ (2.82 eV vs. NHE), which was significantly higher than the oxidation potential of water or (–OH) molecules, which is 2.4 eV. Therefore, the holes were able to react with water (or –OH) molecules and generate hydroxyl radicals (reaction 4) [63]. In contrast, electrons accumulated in the CB of Cu_2_O (−1.48 eV vs. NHE). The electrons were located significantly lower than the reduction potential of oxygen (−0.33 eV), and electrons could react with oxygen to form superoxide radicals (reaction 5) [64]. These main reactive species could further react with RhB dye molecules and decompose into carbon dioxide and water (reaction 6) [65]:

(3) $${\mathrm{TiO}}_{2}/\mathrm{CuO}/{\mathrm{Cu}}_{2}O+hv_{\left(UV-visible\right)}\to {\mathrm{TiO}}_{2}\left(e_{CB}^{-}\;h_{VB}^{+}\right)/\mathrm{CuO}\left(e_{CB}^{-}\;h_{VB}^{+}\right)/{\mathrm{Cu}}_{2}O\left(e_{CB}^{-}\;h_{VB}^{+}\right)$$

(4) $${\mathrm{TiO}}_{2}\left(h_{VB}^{+}\right)+H_{2}O\to \cdot \mathrm{OH}+H^{+}+{\mathrm{TiO}}_{2}$$

(5) $${\mathrm{Cu}}_{2}O\left(e_{CB}^{-}\right)+O_{2}\to \cdot \;O_{2}^{-}+{\mathrm{Cu}}_{2}O$$

(6) $$\cdot \;\mathrm{OH}+\cdot \;O_{2}^{-}+\mathrm{RhB}\to product\;(ex:{\;H}_{2}O+{\mathrm{CO}}_{2})$$

Notably, from among the band alignments proposed for the TiO_2_, Cu_2_O, and CuO, multiple photoinduced charger transfer routes could occur in the ternary TiO_2_–Cu_2_O–CuO composite system. The p-n junctions formed between the n-type TiO_2_ and p-type copper oxides induced an internal electric field at the heterointerfaces, promoting charge separation under irradiation. The stepped-band edge arrangement in the composite system caused multiple Z-scheme transfer routes for the photoinduced charges. This finally resulted in the accumulation of holes at the VB of TiO_2_ and of electrons at the CB of Cu_2_O. A similar Z-scheme carrier movement was also exhibited in a ternary ZnO–Cu_2_O–CuO photocatalyst system [66]. In the report by Wei et al., the composite material TiO_2_–Cu_2_O showed carrier movement with a Z scheme under irradiation [67]. These examples echo the carrier movement mechanism proposed in this work. In addition, the Z-scheme charge transfer in the composite nanorods had an important contribution in preventing the photocorrosion of Cu_2_O. Photocorrosion has been demonstrated in previous work on single-phase Cu_2_O photocatalysts [68]. The Cu_2_O phase coupling with TiO_2_ (TC200) or TiO_2_–CuO (TC300 and TC350) in the composite system effectively guided the photoexcited electrons and holes accumulated in the Cu_2_O and inhibited photocorrosion. Therefore, a stable photocurrent curve could be observed in the previous photoresponse plots. This is supported by work on introducing a protective layer of TiO_2_ in Cu_2_O–CuO heterojunction films to prevent the photocorrosion effect [69]. The multiple charge transfer routes shown in Figure 12 explain the superior photoactive performance of TiO_2_–Cu_2_O–CuO composite systems (TC350 and TC300) among the various TC composite nanorods. Finally, it should be mentioned that TC350 exhibited better photoactivity than that of TC300. This can be attributed to optical absorption and the microstructural differences between the TC300 and TC350. The TC350 exhibited a better light absorption ability than TC300, as revealed by the previous UV-vis absorption analysis, which enhanced the generation efficiency of photoexcited charges in TC350. Moreover, TC350 also had a larger surface particle size in the decorated copper oxide layer in comparison to that of TC300. A larger grain size reduces grain boundaries in the decorated copper oxide layer, resulting in enhanced charge transport [47]. The ternary phase and suitable microstructural and optical properties of TC350 mean that it has excellent photoactivity compared to the other TC composite samples.

## 4. Conclusions

The morphology of copper oxide decorated on a TiO_2_ nanorod template changed from a continuous layer morphology to granular aggregates when the postannealing temperature was varied from 200 to 400 °C. The composite nanorods formed at 350 °C (TC350) exhibited superior photoactive performance compared to the other composite nanorods. The larger particle size resulting from the copper oxide modification in TC350 reduced the grain boundaries in the decorated layer, thereby increasing the charge transport ability. Moreover, the surface-modified Cu_2_O–CuO mixed crystallites on the TiO_2_ template could absorb sunlight more efficiently. These factors enhanced the photoactive performance of the TC350 composite nanorods studied herein. The scavenger tests demonstrated that the Z scheme was the possible carrier movement mechanism in TC350 under irradiation, and that result explains the high photocatalytic degradation ability of TC350 towards organic pollutants. The experimental results obtained herein demonstrate that regulation of the composition phase and microstructure of the modified copper oxide layer through control of the thermal annealing budget for the thin copper layer on TiO_2_ nanorod templates is a promising approach to design copper oxide–TiO_2_ composite nanorods with satisfactory photoactive performance.

## Data Availability

Not applicable.
